# Pregnancy Associated Plasma Protein A2 (PAPP-A2) Affects Bone Size and Shape and Contributes to Natural Variation in Postnatal Growth in Mice

**DOI:** 10.1371/journal.pone.0056260

**Published:** 2013-02-15

**Authors:** Julian Kenneth Christians, Devin Rhys de Zwaan, Sunny Ho Yeung Fung

**Affiliations:** Biological Sciences, Simon Fraser University, Burnaby, British Columbia, Canada; Harvard Medical School, United States of America

## Abstract

Pregnancy associated plasma protein A2 (PAPP-A2) is a protease of insulin-like growth factor binding protein 5 and is receiving increasing attention for its roles in pregnancy and postnatal growth. The goals of the present study were to characterize the effects of PAPP-A2 deletion on bone size and shape in mice at 10 weeks of age, and to determine whether *Pappa2* is the gene responsible for a previously-identified quantitative trait locus (QTL) contributing to natural variation in postnatal growth in mice. Mice homozygous for constitutive PAPP-A2 deletion were lighter than wild-type littermates, and had smaller mandible dimensions and shorter skull, humerus, femur, tibia, pelvic girdle, and tail bone. Furthermore, PAPP-A2 deletion reduced mandible dimensions and the lengths of the skull, femur, pelvic girdle, and tail bone more than would be expected due to the effect on body mass. In addition to its effects on bone size, PAPP-A2 deficiency also altered the shape of the mandible and pelvic girdle, as assessed by geometric morphometrics. Mice homozygous for the PAPP-A2 deletion had less deep mandibles, and pelvic girdles with a more feminine shape. Using a quantitative complementation test, we confirmed that *Pappa2* is responsible for the effects of the previously-identified QTL, demonstrating that natural variation in the *Pappa2* gene contributes to variation in postnatal growth in mice. If similar functional variation in the *Pappa2* gene exists in other species, effects of this variation on the shape of the pelvic girdle might explain the previously-reported associations between *Pappa2* SNPs and developmental dysplasia of the hip in humans, and birthing in cattle.

## Introduction

Insulin-like growth factors (IGFs) play a pivotal role in growth and development [1]. The bioavailability of the IGFs is regulated by six IGF binding proteins (IGFBPs), and the release of IGFs and subsequent IGF signaling is achieved through cleavage of the IGFBPs by proteases [1]. Pregnancy associated plasma protein A (PAPP-A) is an IGFBP protease that was initially identified as a circulating protein of placental origin, but has since been shown to play roles outside of pregnancy in cardiovascular disease, the regulation of bone mineral density, skin healing and aging [2], [3], [4], [5], [6]. Screening of sequence databases for homologs of PAPP-A subsequently identified PAPP-A2, an IGFBP-5 protease that shares 45% amino acid sequence identity with PAPP-A [7]. While less work has examined PAPP-A2, this protein is receiving increasing attention for its roles in human pregnancy [2], [8], reproductive traits in cattle [9], [10], [11], and postnatal growth in mice [12], [13].

The first indication of a role in postnatal growth was our previous work that identified *Pappa2* as a candidate for the gene responsible for the effects of a quantitative trait locus (QTL) affecting adult body size in mice. We refined the location of the QTL to a 1 megabase (MB) region that contains only 4 genes, and found that the QTL affected circulating levels of IGFBP-5, the target of PAPP-A2 [12]. Recently, Conover et al. developed PAPP-A2 knock-out mice which are viable and smaller than wild-type [13]. Because IGFBP-5 is known to play a role in bone metabolism [14], [15], PAPP-A2 is expected to have a particularly pronounced effect on skeletal growth. Deletion of PAPP-A2 leads to shorter femur length, but no effects on bone mineral density were detected [13]. The QTL described above also affects the length of certain bones [16]. In cattle, associations have been found between SNPs in *Pappa2* and difficulty giving birth, potentially through effects on the size or shape of the mother’s pelvis [10]. Others have reported association between developmental dysplasia of the hip and a *Pappa2* SNP in humans [17], again suggesting an effect of PAPP-A2 on bone shape.

The goals of the present study were to further characterize the effects of PAPP-A2 deletion on skeletal characters in mice, including not only bone lengths but also bone shapes, and to determine whether *Pappa2* is the gene responsible for the QTL described above [12]. Although *Pappa2* was previously identified as a candidate for a QTL affecting body size [12], and deletion of PAPP-A2 reduces body size [13], it does not necessarily follow that *Pappa2* is responsible for the effects of the QTL; there might be no functional variation in the *Pappa2* gene between the two wild-type mouse strains studied in the QTL work. To test whether *Pappa2* is the causal gene underlying the QTL, we performed a quantitative complementation test [18], [19], [20]. Establishing whether *Pappa2* is responsible for the effects of the QTL will shed light on the functional significance of naturally-occurring variation in this gene, and will help to identify functionally important coding and regulatory regions.

## Methods

### Ethics Statement

All work was carried out in accordance with the guidelines of the Canadian Council on Animal Care and approved by the SFU University Animal Care Committee (protocol 945B-09).

### PAPP-A2 Deletion Mice

**Figure 1:**
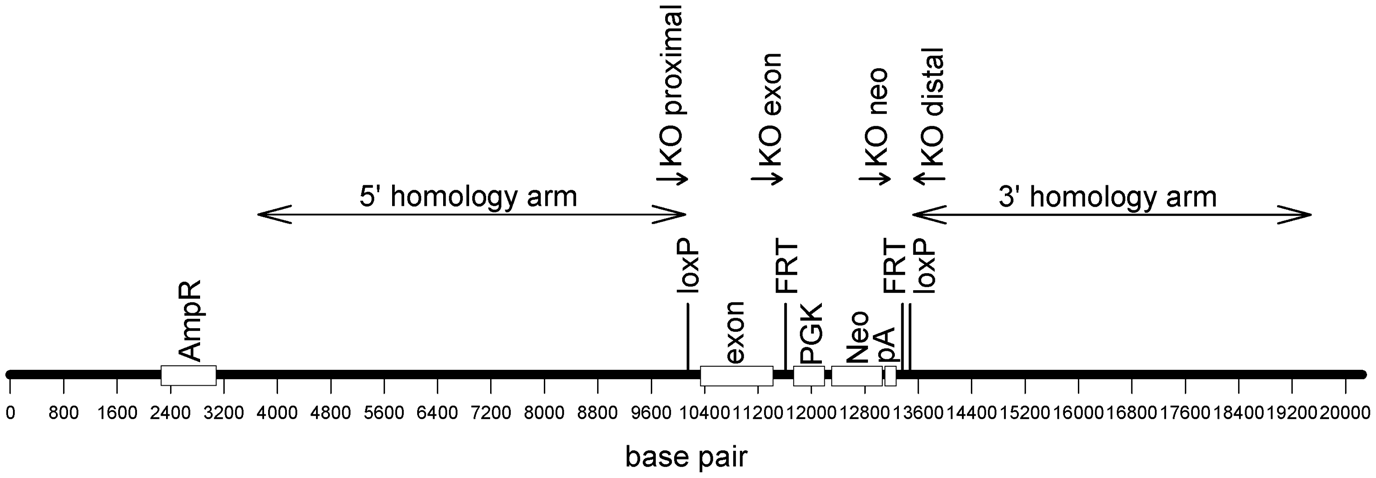
Schematic diagram of the targeting vector used in the generation of PAPP-A2 conditional deletion mice. Shown are the locations of the targeted exon, homology arms, loxP and FRT sites, ampicillin resistance gene (AmpR), phosphoglycerate kinase promoter (PGK), neomycin resistance gene (Neo), bovine growth hormone poly A termination signal (pA), and genotyping primers. The sizes of the primers have been exaggerated for clarity.

Conditional PAPP-A2 deletion mice were generated by Ozgene Pty. Ltd. (Bentley, Australia), i.e., independently of the previous PAPP-A2 deletion study [13]. The targeting vector was constructed using Ozgene’s Pelle plasmid and fragments generated by PCR from C57BL/6 genomic DNA, such that mouse exon 2 (homologous to human exon 3) and a PGK-Neo selection cassette were flanked by LoxP sites (i.e., “floxed”, Fig. 1). Following Cre-mediated deletion of exon 2, splicing of exon 1 to exon 3 is expected to produce an early stop codon. The targeting vector was electroporated into C57BL/6 ES cells and correctly targeted clones were injected into blastocysts for the generation of chimeras. High percentage chimeras were mated with C57BL/6 mice to produce offspring heterozygous for the floxed allele.

Deletion of exon 2 and removal of the Neo cassette were achieved by crossing conditional deletion mice to mice with a C57BL/6 background expressing Cre recombinase (hereafter referred to as Cre) under the control of a human cytomegalovirus minimal promoter (Jackson Laboratory stock number 006054). Removal of the floxed region in the progeny was determined by PCR genotyping (described below). In subsequent experiments (described below), we tested whether Cre itself had an effect on phenotype in preliminary analyses in which we included the presence of Cre (yes/no) as a term in the model. The presence of Cre did not have a significant (P<0.05) effect on body mass or tail length at any age, or on any bone phenotype, in analyses of the effects of PAPP-A2 deletion or the quantitative complementation test, with one exception: the effect of Cre was significant for tail length at 3 weeks of age in the quantitative complementation test, but not in the analyses of the effects of PAPP-A2 deletion. Since the presence of Cre affected only one trait in only one of two experiments, this variable was removed from the model for further analyses.

Mice heterozygous for the constitutive *Pappa2* disruption were paired to produce litters in which all three genotypes were present (i.e., homozygous disruption, heterozygous, and homozygous wild-type). For measuring placental and embryonic weights and PAPP-A2 expression, females were checked for seminal plugs the morning following pairing, and plugged females were euthanized with CO_2_ twelve days later (embryonic day 12.5). We chose to collect females at embryonic day 12.5 to ensure that individual placentae and embryos would be sufficiently large for protein extraction and because PAPP-A2 expression is high at this stage [21], [22]. Placentae and embryos were dissected while immersed in 10× PBS, weighed, and frozen at −80°C for quantification of protein levels by Western blotting. The amniotic sac and/or a piece of embryonic tail was collected for PCR genotyping.

To determine birth weight, females were not checked for plugs, but were instead left with males, checked daily three to five hours after the lights were turned on, and new pups were weighed. We anaesthetized the newborn offspring using isofluorane, and collected 2 mm of tail tissue for genotyping. These pups were also earclipped for genotyping at 3 weeks of age and weighed at 3, 6 and 10 weeks of age. Additional mice were not weighed at birth, but earclipped for genotyping at 3 weeks of age, and mass and tail length were measured at 3, 6 and 10 weeks of age.

### Bone Measurements

Following sacrifice at 10 weeks of age, mice were frozen and thawed at a later date at which time the skin and internal organs were removed and the carcasses were dried and then exposed to dermestid beetles to remove soft tissue. The following bones were measured: mandible length (distance from the tip of the angular process to the anterior edge of the molars, mandible height (from the coronoid process to the tip of the angular process), and the lengths of the skull, humerus, ulna/radius, femur, tibia, pelvic girdle, and a tail bone (the tenth caudal bone counting from the first bone of the sacrum). Where applicable we measured bones from both sides of each animal and calculated the mean. All skeletal dimensions were measured with digital calipers (±0.01 mm) and measurements were performed in triplicate.

### Shape Analysis

In addition to linear size measurements, the shapes of the mandible and pelvic girdle were analyzed. Photographs were taken of the left and right mandible, and left and right pelvic girdles. Landmarks were placed on photos using the tpsDig2 program (tps software package, version 2.16; F. James Rohlf; http://life.bio.sunysb.edu/morph/), with 12 landmarks for mandibles and 14 for the pelvic girdles. The tpsDig2 software was also used to create outline images representing the curvature of the bones, used to visualize differences in bone shape. The tpsUtil program (tps utility software, version 1.49; F. James Rohlf; http://life.bio.sunysb.edu/morph/) was used to form the tps input files.

Procrustes analysis was performed on landmark data using MorphoJ (version 1.05a; C.P. Klingenberg; http://www.flywings.org.uk/MorphoJ_page.htm [23]). The Procrustes analysis uses superimposition to reorient and scale the imported landmark coordinates so they are aligned in the same position and at approximately the same size in order to quantify variation at each landmark coordinate regardless of size or original positioning in the photograph [24]. This analysis also accounts for symmetry between the left and right bones, and left and right bones from a given mouse were subsequently averaged. The Procrustes analysis yields centroid size, a measure of size, and Procrustes coordinates, which provide information on shape, independent of size. Procrustes coordinates were analysed using Principal Component Analysis (PCA) to reduce the dimensionality of the data, i.e., to reduce the multiple distances between coordinates to a smaller number of variables. PCA was performed using the pooled within-group covariance (within litter, sex and genotype). PCA scores, as well as centroid data, were exported from MorphoJ into SAS, Version 9.3 (SAS Institute Inc., Cary, NC) for further analyses. To facilitate comparisons between PAPP-A2 deletion mice and mice from the quantitative complementation test (described below), mice from both experiments were combined for the PCA such that principal component scores describe the same variation in shape for all mice.

### Quantitative Complementation Test

A quantitative complementation test is used to test whether a gene is the causal agent underlying a QTL. The logic is as follows: An individual heterozygous for a knock-out of gene X is crossed with an individual heterozygous for two QTL alleles, producing offspring that vary both for the knock-out and for the QTL alleles. These offspring are measured for the phenotype of interest, i.e., the trait known to be affected by the QTL. If gene X is not the gene responsible for the QTL, and if gene X does not interact with the gene responsible for the QTL, there will be an effect of QTL allele on the phenotype, and there may be an effect of the knock-out on phenotype, but these two effects will be independent of one another, i.e., there will be no statistical interaction between the effect of the knock-out and the effect of the QTL. Conversely, if gene X is the gene responsible, or interacts with the gene responsible for the effect of the QTL, there will be an interaction between the effects of the QTL allele and the effect of the knock-out, i.e., the phenotypic effect of the QTL allele will depend on whether gene X is knocked out or not (see Table 1).

**Table 1:** Predicted phenotypic comparisons in offspring from the quantitative complementation test if *Pappa2* is or is not the quantitative trait gene (QTG), i.e., the gene responsible for the effect of the QTL.

Mouse heterozygous for constitutive *Pappa2* disruption (on C57BL6 background)					
		X			
Mouse heterozygous for C57BL6 and DBA2 QTL alleles (on DBA2 background)					
		↓			
	Offspring of four possible genotypes:disrupted or intact C57BL6 *Pappa2* allele from one parent, and C57BL6 or DBA2 QTL from other parent				
	C57BL6/disrupted		DBA2/disrupted	C57BL6/intact	DBA2/intact
If *Pappa2* is responsible for the effect of the QTL, the genotype at the QTG will be:	C57BL6/disrupted		DBA2/disrupted	C57BL6/C57BL6	DBA2/C57BL6
	**Prediction:** The phenotypic difference between C57BL6/disrupted and DBA2/disrupted will be greater than the phenotypic difference between C57BL6/C57BL6 and DBA2/C57BL6. Little difference is expected between the C57BL6/C57BL6 and DBA2/C57BL6 genotypes because the C57BL6 allele is partially dominant [12].				
If *Pappa2* is not responsible for the effect of the QTL, The genotype at the QTG will therefore be:	C57BL6/C57BL6		DBA2/C57BL6	C57BL6/C57BL6	DBA2/C57BL6
	All offspring will inherit an intact C57BL6 allele at the QTG, even where they inherit a disrupted *Pappa2* allele.**Prediction:** The phenotypic difference between C57BL6/disrupted-*Pappa2* and DBA2/disrupted-*Pappa2* will be equal to the phenotypic difference between C57BL6/intact-*Pappa2* and DBA2/intact-*Pappa2*.				

In previous work, we introgressed the C57BL/6 allele of a chromosome 1 QTL into the DBA/2 background to produce a line segregating for an approximately 1 MB region containing the QTL, including *Pappa2* [12]. This line was at backcross generation 14 at the time of this study. The quantitative complementation test was carried out by crossing mice heterozygous for the QTL allele with mice heterozygous for constitutive *Pappa2* disruption (described above). The offspring inherited either the C57BL/6 allele or DBA/2 allele of the introgressed segment from their QTL line parent, and either the *Pappa2* disruption allele or the wild-type allele from their disruption parent. Outside of the 1 MB QTL region, the offspring all had the same C57BL/6 - DBA/2 heterozygous genetic background. Offspring were earclipped for genotyping at 3 weeks of age, and mass and tail length were measured at 3, 6 and 10 weeks of age.

### Genotyping

Extraction of genomic DNA from ear clips was performed by standard methods. PCR genotyping was used for the determination of (a) *Pappa2* alleles (wild-type vs. disrupted), (b) QTL alleles (DBA/2 vs. C57BL/6) and (c) the presence/absence of the Cre recombinase gene. Genotyping for the *Pappa2* allele used 4 primers at locations shown in Fig. 1. These primers were designed to yield bands of different sizes for the four possible alleles (408 bp for wild-type, 308 bp for deleted, 341 bp for floxed with the Neo selection cassette present, and 547 bp for floxed with the Neo selection cassette absent; only the first two alleles were present in this study). Genotyping of QTL alleles was performed by amplifying two fragments at either end of the QTL region. These fragments were then digested by XbaI which cut only one of the alleles due to a SNP in the restriction site, generating a restriction fragment length polymorphism. The genotyping reactions for Cre included two primer pairs recommended by the Jackson Laboratory: one to amplify a fragment from the Cre transgene and one to amplify a positive control fragment to confirm that the PCR was successful; the positive control primers target an exon of the interleukin 2 gene on chromosome 3. Primer sequences are provided in Table 2.

**Table 2:** Primer sequences.

*Pappa2* allele genotyping	
KO proximal	CAGCAAAGGAAATTTGTGCT
KO distal	ATTCCTCAGCCTCCTCTGAT
KO exon	CTTCGATGATGGAGACTGCT
KO neo	GGATTGGGAAGACAATAGCA
QTL allele genotyping	
Proximal forward	GGTGTTGTGGGTGAGACATT
Proximal reverse	AAACATAAAGTAGACATGCTTCCA
Distal forward	CCTGTCTGACTCACGAAGAAA
Distal reverse	GGTCATGGTCTCTCTTCATACC
Cre genotyping	
Cre forward	GCGGTCTGGCAGTAAAAACTATC
Cre reverse	GTGAAACAGCATTGCTGTCACTT
Positive control forward	CTAGGCCACAGAATTGAAAGATCT
Positive control reverse	GTAGGTGGAAATTCTAGCATCATCC

### Protein Extraction and Western Blotting

Placentae and embryos were homogenized in 2 mL of T-PER™ Tissue Protein Extraction Reagent (PIERCE, Rockford, IL), incubated on ice for 7 minutes and then centrifuged at 10000 g for 5 minutes at 4°C. Complete protease inhibitor cocktail (Roche Applied Sciences) was added to the supernatant, which was then stored at −20°C.

Placental and embryonic samples containing 40 µg of protein were mixed with SDS loading buffer, boiled and run through a 4% stacking and 8% separating polyacrylamide gel for 60 minutes. The gel was equilibrated in transfer buffer and transferred onto a nitrocellulose membrane (Bio-Rad, Hercules, CA). Membranes were blocked for one hour at room temperature in Odyssey Blocking Buffer (Li-Cor Biosciences, Lincoln, Nebraska), incubated for one hour at room temperature (placentae) or overnight at 4°C (embryos) in a solution containing 1∶500 monoclonal mouse anti-actin (CLT9001; Cedarlane, Burlington ON) and 1∶500 polyclonal goat-anti-human PAPP-A2 antibody (AF1668; R&D Systems, Minneapolis, MN) diluted in Odyssey Blocking Buffer (Li-Cor Biosciences, Lincoln, NE) and 0.1% Tween-20 (Sigma, ON, Canada), washed 4 times, and incubated in a solution containing 1∶10000 fluorescently-labelled IRDye 800 anti-goat and IRDye 680 anti-mouse secondary antibodies (Li-Cor Biosciences, Lincoln, NE) diluted in Odyssey Blocking Buffer, 0.1% Tween-20 and 0.1% SDS for 45 minutes with gentle shaking. The membranes were again washed 4 times. Membranes were visualized using the Odyssey infrared imaging system (Li-Cor Biosciences, Lincoln, NE). The two secondary dyes fluoresce at different wavelengths (anti-goat at 800 nm and anti-mouse at 700 nm), so we were able to simultaneously quantify the intensity of the PAPP-A2 band (at approximately 250 kDa) and actin band (at approximately 40 kDa) on the same nitrocellulose membrane.

### Statistical Analyses

All statistical analyses were performed using general linear models (proc GLM) in SAS, Version 9.3 (SAS Institute Inc., Cary, NC).

## Results

### PAPP-A2 Deletion

**Figure 2:**
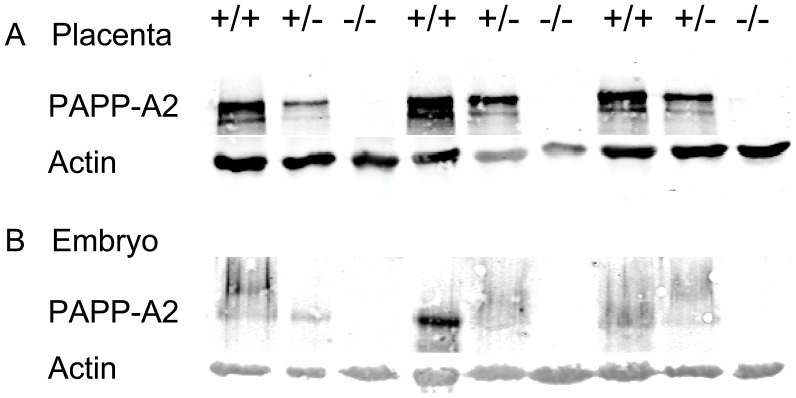
Effects of gene disruption on PAPP-A2 protein. Shown are Western blots of 9 representative (A) placentae and (B) embryos at embryonic day 12.5 from embryos homozygous for wild-type *Pappa2* (+/+), heterozygous (+/−) or homozygous for the disruption (−/−). The nitrocellulose membrane was scanned for fluorescence at 700 and 800 nm simultaneously, with fluorescence at 700 nm corresponding to actin (at approximately 40 kDa) and fluorescence at 800 nm corresponding to PAPP-A2 (at approximately 250 kDa).

Following Cre-mediated excision of the floxed region, bands of the expected size were obtained from PCR (data not shown) and PAPP-A2 protein was not detectable in placentae or embryos homozygous for the disrupted allele, and PAPP-A2 levels in heterozygotes were lower than those in homozygous wild-types (Fig. 2). In crosses between mice heterozygous for the disruption, the offspring genotypes did not differ from the expected Mendelian ratios *in utero*, at birth, or at weaning (Table 3), suggesting no effect of PAPP-A2 deletion on viability.

**Table 3:** Offspring genotype frequencies in crosses between mice heterozygous for the *Pappa2* disruption (−/− = homozygous for *Pappa2* disruption, +/− = heterozygous, +/+ = homozygous for wild-type *Pappa2*).

Stage	−/−	−/+	+/+	P-value^a^
E12.5	3	5	6	0.30
Birth	14	37	11	0.27
Weaning^b^	24	53	18	0.36

a Chi-square test of null hypothesis that genotypes are in 1∶2:1 ratio.

b The sample measured at weaning and into adulthood includes 50 females and 45 males.

### Effects of PAPP-A2 Deletion on Mass and Size

At embryonic day 12.5, there was no detectable effect of PAPP-A2 deletion on placental or embryonic mass (Table 4). There was marginally non-significant variation among genotypes in birth mass (F_2,53_ = 2.54; P = 0.09), with a trend towards lower birth mass in pups homozygous for the PAPP-A2 deletion (Table 4). In MANOVA of size at 3, 6, and 10 weeks of age, the effect of PAPP-A2 genotype was significant for both body mass (Wilks' Lambda F_6,140_ = 9.81; P = 0.0001) and tail length (Wilks' Lambda F_6,38_ = 8.75; P = 0.0001), and so further single variable tests were performed. The effect of PAPP-A2 deletion generally did not depend on sex; the only significant genotype by sex interaction (F_2,71_ = 3.30; P = 0.04) was for mass at 6 weeks. Removing the genotype by sex interaction term from the model, variation among genotypes was significant for body mass at 3 weeks and for body mass and tail length at 6, and 10 weeks of age (Table 4); the effect on 3 week tail length was marginally non-significant (F_2,24_ = 2.82; P = 0.08). For all traits, mice homozygous for the PAPP-A2 deletion were smaller, while there was no evidence of a difference between heterozygotes and homozygous wild-types (Table 4).

**Table 4:** Phenotypes in offspring homozygous for *Pappa2* disruption (−/−), heterozygous (+/−), or homozygous for wild-type *Pappa2* (+/+), from crosses between mice heterozygous for the *Pappa2* disruption.

	−/−	−/+	+/+	P-value^a^
Placental massat E12.5 (g)	0.09±0.01	0.10±0.01	0.11±0.01	0.55^b^
Embryonic massat E12.5 (g)	0.14±0.01	0.14±0.01	0.14±0.01	1.00^b^
Birth mass (g)	1.25±0.03	1.31±0.02	1.32±0.03	0.09^c^
3 week mass (g)	7.25±0.21	8.14±0.14	8.20±0.24	0.002^d^
3 week tail length (cm)	6.06±0.11	6.32±0.07	6.40±0.11	0.08^d^
6 week mass (g)	14.79±0.40	17.41±0.26	17.60±0.46	0.0001^d^
6 week tail length (cm)	6.96±0.10	7.41±0.06	7.35±0.12	0.004^d^
10 week mass (g)	18.93±0.31	21.91±0.19	21.95±0.36	0.0001^d^
10 week tail length (cm)	7.65±0.06	8.28±0.03	8.23±0.07	0.0001^d^
Mandible length (mm)	7.87±0.03	8.29±0.02	8.29±0.04	0.0001^d^
Mandible height (mm)	5.89±0.03	6.34±0.02	6.34±0.04	0.0001^d^
Skull (mm)	21.43±0.09	22.23±0.07	22.07±0.11	0.0001^d^
Humerus (mm)	11.24±0.06	11.55±0.04	11.49±0.07	0.0003^d^
Ulna/radius (mm)	13.07±0.08	13.29±0.06	13.23±0.09	0.07^d^
Femur (mm)	14.17±0.07	14.78±0.05	14.66±0.08	0.0001^d^
Tibia (mm)	16.42±0.07	16.83±0.05	16.70±0.09	0.0003^d^
Pelvic girdle (mm)	16.42±0.14	17.57±0.10	17.48±0.17	0.0001^d^
Tail bone (mm)	3.34±0.05	3.72±0.03	3.72±0.05	0.0001^d^

Values are least squares means ± standard error.

a P-values are provided for the test of whether there is significant variation among the three genotypes.

b Sample sizes are as in Table 3. Placentae and embryos were collected from two pregnant females. Genotype and female were included as terms in the model.

c Sample sizes are as in Table 3. Pups from 7 litters were measured. Genotype and litter were included as terms in the model.

d Sample sizes for body mass are approximately as in Table 3, although are lower in some cases because of missed measurements. Sample sizes for bone measurements are smaller (total N = 73) because some mice were used for further breeding and so were not sacrificed at 10 weeks of age. Offspring from 11 litters were measured. Sample sizes for tail length are substantially smaller (total N = 27–30) because this trait could not be measured in individuals for which birth mass was obtained since the tip of the tail was collected for genotyping. Genotype, litter and sex were included as terms in the model.

In analyses of the linear dimensions of bones, MANOVA identified a significant effect of genotype on bones of the skull (skull and mandible dimensions; Wilks' Lambda F_6,114_ = 17.81; P = 0.0001) and the long bones (humerus, radius/ulna, femur and tibia; Wilks' Lambda F_8,114_ = 6.26; P = 0.0001), and so further single variable tests were performed. As with body mass and tail length, the effect of PAPP-A2 deletion did not depend on sex; genotype by sex interaction was not significant for any trait and so was removed from the model. Consistent with the highly significant effect on 10 week body mass and tail length, PAPP-A2 deletion had a significant effect on mandible dimensions and the lengths of the skull, humerus, femur, tibia, pelvic girdle, and tail bone (Table 4); the effect on the length of the ulna/radius was marginally non-significant (F_2,60_ = 2.71; P = 0.08). In all cases, mice homozygous for the PAPP-A2 deletion had shorter bones, while bone size was similar in heterozygotes and homozygous wild-types. To determine whether effects on bone dimensions were due to a general effect on size, or whether certain bones were affected disproportionately, we repeated the analysis while controlling for 10 week body mass (by including body mass as a covariate in the model). Effects of genotype on the humerus, ulna/radius and tibia were not significant when controlling for body mass (data not shown). However, there remained significant effects on mandible dimensions and the lengths of the skull, femur, pelvic girdle, and tail bone with smaller bones in homozygous knock-out mice, even when body mass was included as a covariate (data not shown), i.e., the length of these bones was reduced more than would be expected given the effect of PAPP-A2 deletion on body mass.

In contrast to the effects of PAPP-A2 deletion on bone lengths, there was no effect of *Pappa2* genotype on the dry masses of the stomach, spleen, pancreas, kidneys, liver, lungs or heart (MANOVA Wilks' Lambda F_16,100_ = 1.42; P = 0.15).

### Effects of PAPP-A2 Deletion on Bone Shape

The Procrustes analysis used to remove variation in position, orientation and size from bone landmark data yielded centroid size, which is a measure of overall bone size. Consistent with the linear measures described above, deletion of PAPP-A2 had a highly significant effect on the centroid size of both the mandible and pelvic girdle (P<0.0001 in both cases), with mice homozygous for *Pappa2* disruption having smaller bones, and heterozygotes and homozygous wild-type mice having bones of similar size.

**Figure 3:**
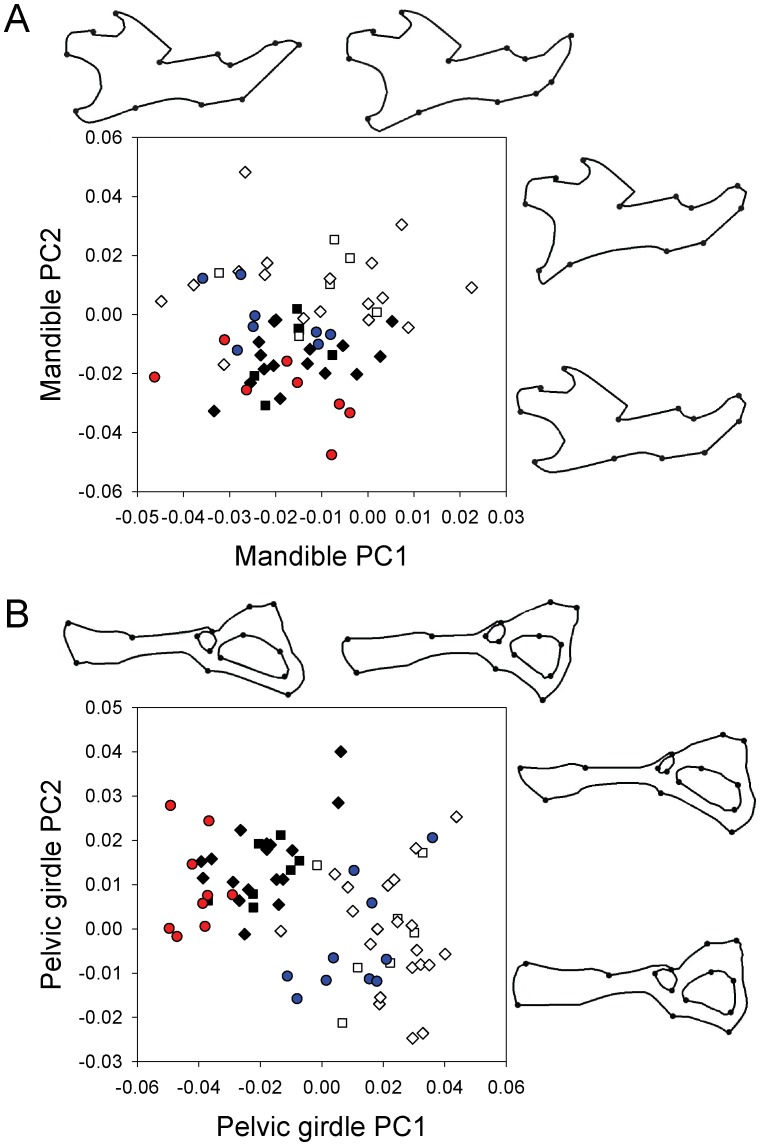
Effects of gene disruption on mandible and pelvic girdle shape. In the scatterplots of the first and second principal components of (A) mandible landmark data and (B) pelvic girdle landmark data, black/red symbols are females, open/blue symbols are males, squares are homozygous wild-type, diamonds are heterozygotes and circles are homozygous *Pappa2* disruption. To highlight the effects of disruption, symbols for homozygote knock-out females are red and those for homozygote knock-out males are blue. Outlines above the graph illustrate variation in shape measured by the first principal component, and represent extremes in shape (PC1 = −0.1 and 0.1) to facilitate visualization. Outlines to the right of the graph illustrate variation in the second principal component. Dots on the outlines indicate the location of landmarks. Note that analyses are carried out on the locations of landmarks, and outlines are drawn only to visualize changes in shape; outlines should therefore be interpreted with caution. Because of broken, damaged or missing bones, sample sizes were reduced compared to analyses of body mass, and a total of 61 mandibles and 71 pelvic girdles were analysed.

In Principal Component Analysis of the Procrustes coordinates, the first two principal components explained 47% and 34% of the variation in size-independent shape of mandibles and pelvic girdles, respectively; subsequent principal components each explained less than 12% of the variation. Plotting the second principal component (PC2) against the first (PC1) illustrates differences in bone shape between mice homozygous for *Pappa2* disruption and those with at least one functional copy of *Pappa2*, as well as differences between sexes (Figs. 3A and 3B). Including sex and litter as terms in the model, there was significant variation among *Pappa2* genotypes in mandible PC2 (F_2,50_ = 8.70; P = 0.0006) but not PC1 (F_2,50_ = 0.87; P = 0.43). As with measures of size, shape in heterozygous mice was much more similar to homozygous wild-types than to mice homozygous for *Pappa2* disruption. In mandibles, PC2 includes variation in the distance between the tips of the angular and coronoid processes (Fig. 3A). Mice homozygous for the *Pappa2* disruption have more negative values of PC2 (Fig. 3A), which indicates a smaller distance between the tips of the angular and coronoid processes, i.e., a less deep bone.

There was also significant variation among *Pappa2* genotypes in pelvic girdle PC1 (F_2,58_ = 9.26; P = 0.0003), but not pelvic girdle PC2 (F_2,58_ = 0.13; P = 0.88), including sex and litter as terms in the model. As with above, shape in heterozygous mice was much more similar to homozygous wild-types than to mice homozygous for *Pappa2* disruption. Pelvic girdle PC1 largely reflects variation in the shape of the ischium, as well as the length of the pubis and, to a lesser extent, the width of the end of the ilium (Fig. 3B). This captured the sexual dimorphism in pelvic girdle shape, with little overlap in PC1 between males and females. Mice homozygous for the *Pappa2* disruption had significantly lower PC1 scores, suggesting a more “female” shape, i.e., male knock-outs were more similar to females than were other males, and female knock-outs had a more extreme female shape than the other females. Because there was such a striking sexual dimorphism in pelvic girdle shape, we repeated the Principal Components analysis with females only, so that variation between the sexes would not influence the PC scores. The difference in PC1 between females homozygous for the disruption and females with at least one functional copy of *Pappa2* remained significant and the variation in shape associated with PC1 was very similar to that depicted in Fig. 3B (data not shown).

### Quantitative Complementation Test

**Figure 4:**
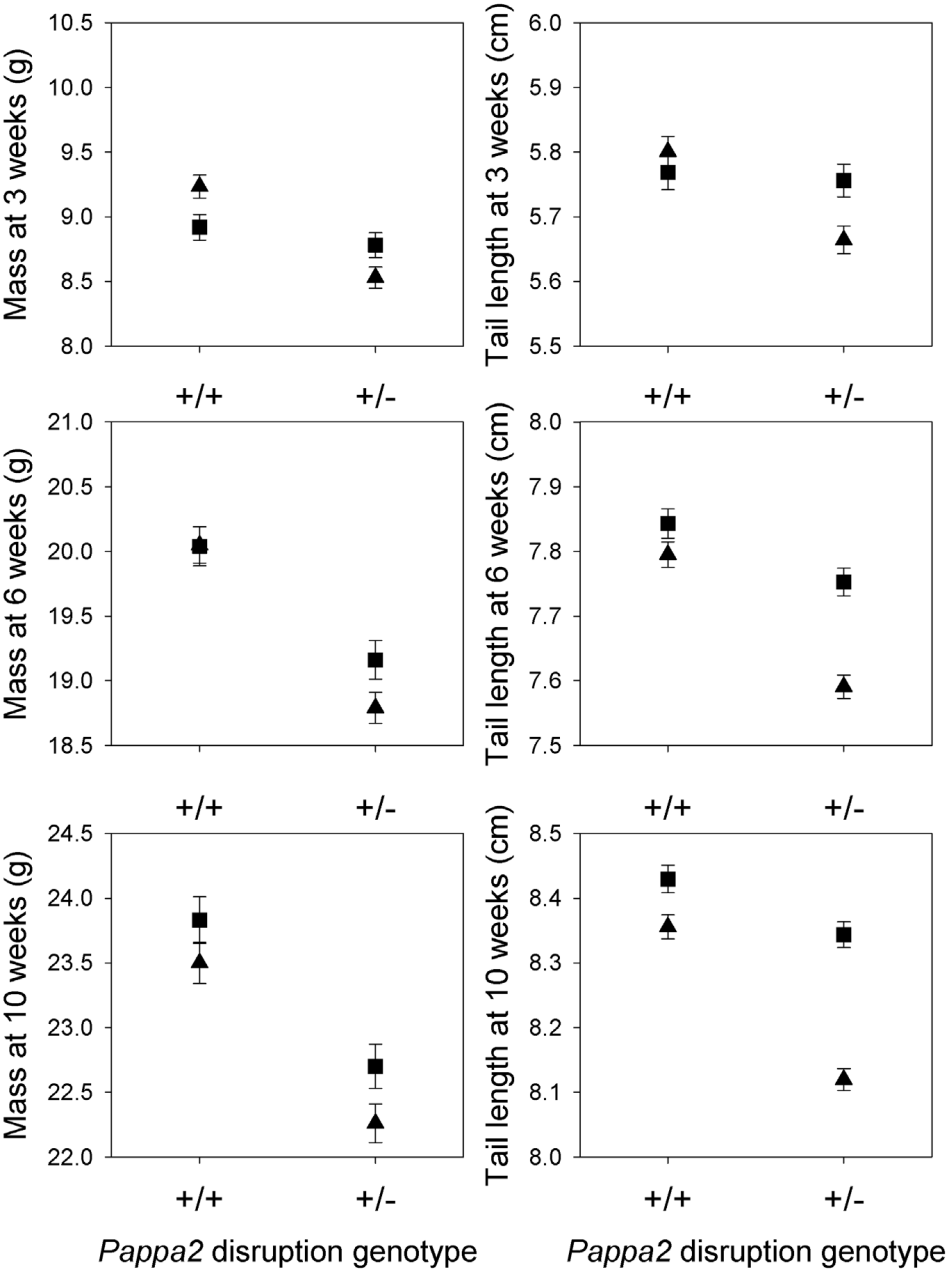
Quantitative complementation test. Body mass and tail length at 3, 6 and 10 weeks of age are shown for mice homozygous for wild-type *Pappa2* (+/+) or heterozygous for the *Pappa2* disruption (+/−) and homozygous for the C57BL6 QTL allele (squares) or heterozygous for the DBA2 and C57BL6 QTL alleles (triangles). In all cases, the difference between QTL alleles is greater for +/− mice than for +/+ mice, consistent with what is predicted if *Pappa2* is the gene responsible for the QTL. The total sample size for the quantitative complementation test was 243 (133 females and 110 males), although is slightly lower for some traits due to missed measurements.

A quantitative complementation test was performed to test whether *Pappa2* is the gene responsible for a previously-described QTL affecting body size. The prediction is that, if *Pappa2* is the gene responsible for the effect of the QTL, the phenotypic difference between QTL genotypes will be greater in mice who inherited a disrupted *Pappa2* allele than in mice who inherited a wild-type *Pappa2* allele from their heterozygous disruption parent (Table 1). The mean values fit this prediction for tail length and body mass at 3, 6, and 10 weeks of age (Fig. 4), with mice inheriting a DBA/2 allele and a disrupted *Pappa2* allele having the lowest values. In MANOVA, the statistical interaction between *Pappa2* disruption genotype and QTL genotype was significant for body mass (Wilks' Lambda F_3,200_ = 3.60; P = 0.014) and tail length (Wilks' Lambda F_3,198_ = 6.00; P = 0.0006) at 3, 6 and 10 weeks. However, in single variable tests, the interaction was significant only for tail length at 3, 6 and 10 weeks (F_1,201_ = 6.60; P = 0.01, F_1,201_ = 7.93; P = 0.005, and; F_1,200_ = 15.45; P = 0.0001, respectively) and for body mass at 3 weeks (F_1,203_ = 9.71; P = 0.002), but not at 6 or 10 weeks (F_1,203_ = 1.81; P = 0.18 and F_1,202_ = 0.11; P = 0.74, respectively). Litter and sex were also included as terms in the model in these analyses. The quantitative complementation test therefore established that *Pappa2* is responsible for at least part of the effect of the QTL.

The interaction between *Pappa2* disruption genotype and QTL genotype was marginally non-significant for the bones of the skull (skull and mandible dimensions; MANOVA Wilks' Lambda F_3,194_ = 2.22; P = 0.09) and not significant for the long bones (humerus, radius/ulna, femur and tibia; MANOVA Wilks' Lambda F_4,194_ = 0.20; P = 0.94). In single variable analyses, the quantitative complementation test was significant for mandible length (F_1,198_ = 5.66; P = 0.02), the length of the pelvic girdle (F_1,198_ = 3.90; P = 0.05) and was marginally non-significant for mandible height (F_1,196_ = 3.60; P = 0.06), and the centroid sizes of the mandible (F_1,189_ = 3.55; P = 0.06) and pelvic girdle (F_1,201_ = 2.83; P = 0.09), but not significant for any other linear bone measurements or shape measurements (data not shown). While the interaction was not significant, the expected trend (see Table 1) was observed for all linear bone measurements.

## Discussion

We have shown that deletion of PAPP-A2 reduces the lengths of some bones beyond what is expected due to reduced body size, and also affects bone shape. In particular, we found changes in pelvic girdle shape, providing a potential explanation for the previously-reported associations between *Pappa2* SNPs and developmental dysplasia of the hip in humans [17] and birthing in cattle [10]. We found a substantial sexual dimorphism in pelvic girdle shape, consistent with previous work [25], and the pelvic girdle of mice homozygous for the *Pappa2* disruption had a more feminine shape, in addition to being disproportionately small for a given body size. Natural variation in the *Pappa2* gene could therefore increase the risk of birthing complications through a reduction in PAPP-A2 expression or activity leading to reduced pelvic girdle size and/or to an extreme female shape that was suboptimal for giving birth. Alternatively, a SNP that increased PAPP-A2 activity or expression might lead to a pelvic girdle with a more masculine shape, which could also cause difficulties during birth.

Deletion of PAPP-A2 also affected the shape of the mandible, with mice homozygous for the disruption having a smaller distance between the tips of the angular and coronoid processes, i.e., a less deep bone. We therefore hypothesize that natural variation in *Pappa2* is a potential source of variation for evolutionary adaptations in mandible shape, e.g., in response to digging behaviour [26] or diet [27], [28]. For example, shallower mandibles have been associated with a more omnivorous diet, whereas deeper, more robust mandibles have been associated with diets which required more powerful mastication [27].

Unlike the study by Conover et al. [13], we did not find that the effects of PAPP-A2 deletion differed between the sexes. Despite high PAPP-A2 expression in the placenta [22], we did not find prenatal effects of PAPP-A2 deletion, although there was a non-significant trend towards lower birth mass in pups homozygous for the *Pappa2* disruption. A lack of effect on birth mass is consistent with the previous study of PAPP-A2 knock-outs [13], as well as our previous work examining variation in placental PAPP-A2 expression between QTL alleles [21].

The specific mechanisms through which the deletion of PAPP-A2 affects bone size and shape requires further study, but we expect that deletion of PAPP-A2 will lead to a reduction in IGFBP-5 proteolytic activity and consequently higher levels of intact IGFBP-5 and lower free IGF levels, leading to reduced growth. In addition to the effects of IGFBP-5 on IGF availability, IGFBP-5 may also have IGF-independent effects [15]. Because the IGFBPs are thought to have important local actions in bone [14], [15], the consequences of PAPP-A2 deletion may be due to local effects, and so PAPP-A2 deletion would not necessarily be expected to influence circulating levels of IGFBP-5 or IGFs.

In addition to characterizing the effects of PAPP-A2 deletion on skeletal phenotypes, the quantitative complementation test established that *Pappa2* is responsible for at least some of the effect of a previously-described QTL [12]. The quantitative complementation test showed significant interactions for tail length at 3, 6 and 10 weeks and body mass at 3 weeks, and was significant or marginally non-significant for measures of mandible and pelvic girdle size, but was not significant for body mass at 6 or 10 weeks or the sizes of any other bones. These results are consistent with previous observations of the effects of the QTL. Previously, we have found that the effects of the QTL on body mass are smaller than those on tail length [29], [30]. Furthermore, we have only found effects of the QTL on the dimensions of the mandible and tail bone, but not other long bones, although this earlier study used a different measure of the pelvic girdle [16]. It is not clear whether the lack of significance in the quantitative complementation tests for bones other than mandible and pelvic girdle represents a real lack of effect of the QTL or low statistical power. The quantitative complementation test therefore demonstrates that natural variation in the *Pappa2* gene, which exists between the inbred lines of mice used in our previous QTL mapping work, contributes to variation in at least some aspects of body size in mice. This is one of the first instances in which the gene responsible for a QTL affecting growth has been identified. This QTL is due to variation between the C57BL/6 and DBA/2 alleles, and both of these strains have been sequenced extensively. Many if not all of the candidate variants between the alleles are therefore already known, and can be used to investigate functionally important coding and regulatory regions. Although it is not clear whether the QTL is due to regulatory or coding sequence variation, we have previously found differences in PAPP-A2 expression levels between QTL genotypes [21].

In conclusion, our results show that natural variation in the *Pappa2* gene can contribute to variation in postnatal growth. If similar functional variation in the *Pappa2* gene exists in other species, effects of this variation on the shape of the pelvic girdle could potentially explain the previously-reported associations between SNPs in *Pappa2* and pelvic bone-related phenotypes such as hip dysplasia and birthing ease.
